# Identification by Matrix-Assisted Laser Desorption Ionization–Time of Flight Mass Spectrometry and Antifungal Susceptibility Testing of Non-*Aspergillus* Molds

**DOI:** 10.3389/fmicb.2020.00922

**Published:** 2020-06-03

**Authors:** Ying Li, He Wang, Xin Hou, Jing-Jing Huang, Pei-Chang Wang, Ying-Chun Xu

**Affiliations:** ^1^Department of Clinical Laboratory, Xuanwu Hospital, Capital Medical University, Beijing, China; ^2^Department of Clinical Laboratory, Peking Union Medical College Hospital, Chinese Academy of Medical Sciences, Beijing, China; ^3^Beijing Key Laboratory for Mechanisms Research and Precision Diagnosis of Invasive Fungal Diseases, Beijing, China

**Keywords:** antifungal susceptibility testing, identification, MALDI-TOF MS, non-*Aspergillus* molds, Sensititre YeastOne

## Abstract

Non-*Aspergillus* molds including Mucorales, *Fusarium*, and *Scedosporium*, etc. are emerging pathogens leading to higher mortality in immunocompromised patients. Fifty-two isolates of genetically confirmed non-*Aspergillus* molds representing 16 species from 8 genera were collected to evaluate the performance of the Bruker matrix-assisted laser desorption ionization–time of flight mass spectrometry (MALDI-TOF MS) in identification of non-*Aspergillus* molds. Antifungal susceptibilities were determined through the Clinical & Laboratory Standards Institute (CLSI) M38-A2 broth microdilution method and the Sensititre YeastOne colorimetric method. Bruker MALDI-TOF MS identified 57.7% (30/52) of isolates cultured in broth and 15.4% (8/52) of isolates cultured on solid agar media to the species level, respectively, according to standard interpretation criteria. Lowering the species level cut-off value (COV) from ≥2.0 to ≥1.7 could improve the MALDI-TOF MS species-level identification rate to 67.3% (38/52) for isolates cultured on solid media, with a slight increase of false identification rate of 2.6% (1/38). Amphotericin B was the most *in vitro* fungistatic-active agent for 98.1% (51/52) of the tested non-*Aspergillus* molds, with minimum inhibitory concentrations (MICs) of ≤2 μg/mL. The susceptibilities to triazoles varied, with MICs of 0.12 to >16 μg/mL among different species of non-*Aspergillus* molds. The correlation between the CLSI method and Sensititre YeastOne on antifungal susceptibility testing of non-*Aspergillus* molds was good, with essential agreement (EA) rates of >90% for triazoles and echinocandins except amphotericin B, which had a lower EA rate of 84.6%. In conclusion, a favorable performance of the Bruker MALDI-TOF MS in identification of clinical non-*Aspergillus* isolates directly inoculated on solid agar media could be achieved with the adoption of alternative interpretation criteria. Antifungal susceptibility testing is important for non-*Aspergillus* molds, especially when information on triazole susceptibility is required, and the Sensititre YeastOne is a practical and reliable method to determine antifungal susceptibilities of non-*Aspergillus* molds.

## Introduction

In recent years, opportunistic filamentous fungi have come to pose a serious threat to immunocompromised patients with AIDS, leukemia, organ transplantation, etc. Even though *Aspergillus* remains the leading pathogen in invasive mold infections, infections due to non-*Aspergillus* molds are increasingly reported in the clinical setting, with higher mortality rates. Mucorales and *Fusarium* are considered the most frequently clinically encountered non-*Aspergillus* mold pathogens, while other less common species such as *Scedosporium*, *Paecilomyces*, and the deeply thought contaminating pathogen from environment, *Penicillium*, are raising serious attention as a growing number of clinical infections due to those rare species have been reported (Slavin et al., 2015; Douglas et al., 2016; Lass-Flörl and Cuenca-Estrella, 2017). Morphological identification of non-*Aspergillus* molds is usually less effective, especially when species-level information is desired. Molecular sequencing analysis based on informative DNA targets such as the internal transcribed spacer (ITS) region could be competent in identification of non-*Aspergillus* molds, but the time-consuming aspect and need for specialized molecular biological equipment limited its wide use in clinical microbiology laboratories. Matrix-assisted laser desorption ionization—time of flight mass spectrometry (MALDI-TOF MS) has shown excellent performance in identification of bacteria and yeasts (Singhal et al., 2015; Cassagne et al., 2016), and could also achieve accurate identification of *Aspergillus* with in-house databases and adjusted interpretation criteria (Li et al., 2017; Normand et al., 2017b). However, the practicability of MALDI-TOF MS in identification of non-*Aspergillus* molds is somehow restricted and relevant studies are much fewer compared with those on *Aspergillus*.

Non-*Aspergillus* molds demonstrated diverse susceptibilities against the clinically available antifungal agents, and the susceptibility profiles of those molds cannot always be inferred directly from their species information, even a large number of Mucorales and *Fusarium* isolates are notoriously resistant to multiple antifungals including triazoles and echinocandins (Wiederhold, 2017). Although the Clinical & Laboratory Standards Institute (CLSI) and the European Committee on Antimicrobial Susceptibility Testing (EUCAST) have both put forward reference methods, namely CLSI M38-A2 and E.DEF 9.3.1 documents, against conidia-forming molds, those methods have not usually been employed in clinical laboratories because of the shortcomings of complicated operation and difficult endpoint reading. The Sensititre YeastOne YO10 system is a commercial kit of antifungal susceptibility testing based on the broth microdilution method for yeasts and *Aspergillus*, and good performances have been achieved, especially for yeasts (Posteraro et al., 2015; Mello et al., 2017). Although non-*Aspergillus* molds are not listed as detectable species in the Sensititre YeastOne manual, several studies have applied Sensititre YeastOne to determine the antifungal susceptibilities of non-*Aspergillus* molds because it shares the same methodology as the CLSI M38-A2 method expect for its colorimetric endpoint reading feature (Carrillo-Muñoz et al., 2006; Patel et al., 2007; Halliday et al., 2016). Owing to the limited isolates and antifungal drugs involved in previous studies, there is no widely accepted conclusion about the practicability of Sensititre YeastOne on antifugal susceptibility testing of non-*Aspergillus* molds.

In this study, we evaluated the performance of the Bruker MALDI-TOF MS in identification of the clinically important non-*Aspergillus* molds grown under different culture conditions. Further antifungal susceptibility profiles of these isolates were determined through the reference broth microdilution method and the Sensititre YeastOne system, and the capability of the use of Sensititre YeastOne for non-*Aspergillus* molds was assessed.

## Materials and Methods

### Isolates

A total of 52 isolates of non-*Aspergillus* molds isolated from various clinical specimens including respiratory tract (*n* = 34, 65.4%), pus (*n* = 5, 9.6%), wound swabs (*n* = 4, 7.7%), conjunctival congestion secretions (*n* = 2, 3.8%), ascitic fluid (*n* = 2, 3.8%), urine (*n* = 2, 3.8%), blood culture (*n* = 1, 1.9%), biopsy specimens (*n* = 1, 1.9%), and pancreatic drainage fluid (*n* = 1, 1.9%) were collected at Peking Union Medical College Hospital from January 2016 to December 2017. All isolates were initially identified with morphological method and stored as spore suspensions in sterile 20% glycerol at −80°C.

### Molecular Sequencing Identification

Isolates were inoculated on Sabouraud dextrose agar (SDA) plates (Oxoid, United Kingdom) at 28°C for 2–7 days and mycelia were collected for genomic DNA extraction. The internal transcribed spacer (ITS) region was employed as the primary sequencing gene for all isolates. As to *Fusarium*, the eukaryotic translation elongation factor 1α (*EF-1*α) gene was used additionally for species level identification (Wang et al., 2011). Sequence data were analyzed against the National Center for Biotechnology Information (NCBI)^1^ or Mycobank^2^ database, and sequence-based species identification was defined by 99% sequence similarity with 95% query coverage.

### MALDI-TOF MS Identification

Isolates were inoculated on SDA plates at 28°C for 2–5 days until the colonies reached a size of about 2 cm diameter. The front mycelia were collected with a sterile inoculating loop and then transferred to 1.5-mL Eppendorf tubes containing 300 μL of distilled water and 900 μL of ethanol. The suspension was centrifuged at 16,000 × g for 3 min, and the pellet was dried at room temperature for 10 min. The pellet was then resuspended in 50 μL of 70% formic acid (FA) for 5 min and an equal volume of acetonitrile was then added. Samples were subsequently centrifuged at 16,000 × g for 2 min. One microliter of supernatant was transferred to the polished steel MTP 384 target plate (Bruker Daltonik, GmbH) and allowed to dry at room temperature before being overlaid with 1 μL of a saturated a-cyano-4-hydroxycinnamic acid (HCCA) matrix solution (Bruker Daltonik, GmbH). Meanwhile, spore suspensions with an approximate size of 1 × 10^6^ colony-forming units (CFUs) from the colonies on SDA plates were reinoculated with 10 mL of Sabouraud dextrose broth (SDB) at 28°C for approximately 1 day in a rotator as the Bruker standard operating procedure suggested. Then 1 mL of fungus-containing medium was transferred to a 1.5-mL Eppendorf tube and centrifuged at 16,000 × g for 3 min, and the pellet was washed twice with 1 mL of deionized water. The remaining operation was identical to the aforementioned standard FA–acetonitrile extraction method applied in this study. The acquisition and analysis of mass spectra were performed by a Bruker Autoflex^TM^ LT mass spectrometer using the MALDI Biotyper software package (version 3.1, Bruker Daltonik, GmbH), and the Filamentous Fungi Library 1.0 (Bruker Daltonik, GmbH) was used as the analysis database. Identification scores of ≥2.0 indicated species-level identification, scores of 1.7–1.999 indicated genus-level identification, and scores of <1.7 were considered unreliable according to the Bruker manual. We also evaluated the performance of the Bruker MALDI-TOF MS in non-*Aspergillus* identification by lowering the species-level identification cut-off value (COV) from ≥2.0 to ≥1.7 and the genus-level identification COV from 1.7–1.999 to 1.4–1.699 followed by reinterpretation of the identification records.

### Antifungal Susceptibility Testing

Susceptibility testing was carried out using the broth microdilution method according to the CLSI M38-A2 document. The drugs tested were amphotericin B [AMB; National Institute for Food and Drug Control (NIFDC), China], itraconazole (ITR; NIFDC, China), voriconazole (VOR; NIFDC, China), posaconazole (POS; Merck, United States), caspofungin (CAS; Merck, United States), and micafungin (MF; Astellas, Japan). Drug concentration ranges for AMB, ITR, VOR, and POS were 0.015–16 μg/mL; and 0.008–8 μg/mL for CAS and MF. *Candida parapsilosis* (ATCC 22019) and *Candida krusei* (ATCC 6258) were included as the quality control strains for each run as positive controls of the antifungals’ potency. Minimum effective concentrations (MECs) of echinocandins and minimum inhibitory concentrations (MICs) of the other tested agents were taken visually after 24 h for Zygomycetes; for *Fusarium* and the remaining species, 48-h or 72-h reading were needed as the CLSI protocol recommended. The ranges of MICs or MECs were calculated for each species–antifungal agent combination. We also evaluated the practicability of the Sensititre YeastOne YO10 panel in antifungal susceptibility testing of non-*Aspergillus* molds, and the agreement between these two methods in susceptibility determination of non-*Aspergillus* molds was analyzed. Sensititre YeastOne testing was carried out according to the manual instructions. For AMB and triazoles, the colorimetric MICs were taken as the lowest concentration well when the antifungal solution changed from red (growth) to blue (100% growth inhibition) at 24 h or 48 h depending on the respective species. MECs of echinocandins were defined as the lowest drug concentration that allowed the growth of small, rounded, and degenerated hyphae at 24 h or 48 h regardless of the solution color change in the wells.

Essential agreement (EA) between the Sensititre YeastOne and CLSI M38-A2 on susceptibility determination of non-*Aspergillus* molds was considered when the MICs/MECs obtained with the two methods fell within 2 dilutions of the twofold dilution scheme. EA values of >90% were considered acceptable (Lamoth and Alexander, 2015).

### Statistical Analysis

Statistical calculations were done using IBM SPSS statistics software, version 19 (SPSS Inc., Chicago, IL, United States). Differences between the tested COVs for MALDI-TOF MS identification were evaluated using the Wilcoxon signed-rank test, and *P* < 0.05 was considered statistically significant (Schulthess et al., 2014).

## Results

### Species Information for Isolates

Fifty-two isolates of non-*Aspergillus* molds including Mucorales (*n* = 23, 10 species), *Fusarium* spp. (*n* = 21, 3 species), *Paecilomyces* spp. (*n* = 4, 2 species), and *Scedosporium apiospermum* (*n* = 4) were revealed by molecular sequencing analysis. The ITS sequences of isolates of interest obtained included in the study were deposited on GenBank (accession numbers MT254752, MT254823, MT254824, MT259026–MT259029, and MT279277–MT279300).

### MALDI-TOF MS Identification

Applying the standard interpretation criteria recommended by the manufacturer, the Bruker MALDI-TOF MS identified 57.7% (30/52) of isolates cultured in SDB to the species level with scores of ≥2.0 and 34.6% (18/52) to the genus level with scores of 1.7–1.999, whereas for isolates cultured on SDA plates, only 15.4% (8/52) of isolates could achieve species-level identification and 57.7% (30/52) to genus level. Correct identification rates at the species level could be reached to 88.5% (46/52) for SDB-cultured isolates and 76.9% (40/52) for SDA-cultured isolates by the Bruker MALDI-TOF MS regardless of the score values obtained after comparison with the sequencing identification results. There were 11.5% (6/52) of SDB-cultured isolates and 21.1% (11/52) of SDA-cultured isolates identified correctly at the genus level by Bruker MALDI-TOF MS, and most of them had lower scores of <1.7. Only one isolate of *Mucor hiemalis* cultured on SDA was totally misidentified at the genus level, with a score of 1.316. After lowering the interpretation criteria, i.e., the species-level COV from scores of ≥2.0 to ≥1.7, the species-level identification rates increased significantly to 92.3% (48/52) (*Z* = −3.169, *P* = 0.002) and 73.1% (38/52) (*Z* = −3.224, *P* = 0.001) for SDB- and SDA-cultured isolates, respectively. For isolates directly grown on SDA plates, the reanalyzed species level identification rate with the lowered COV was comparable to the rate of isolates cultured in broth with the original criteria, namely the Bruker recommended procedure (73.1% vs. 57.7%). Unfortunately, the adjusted COV also brought species-level false identification cases; 6.3% (3/48) of isolates cultured in SDB and 2.6% (1/38) of isolates on SDA with scores of ≥1.7 turned out to be falsely identified at the species level but with the correct genus information, and most of those misidentified cases were from species without reference spectra in the Bruker database (Table 1).

**TABLE 1:** Identification of 52 isolates of non-*Aspergillus* molds by the Bruker MALDI-TOF MS system based on different culture conditions and interpretation criteria for results.

Species	Isolates	Broth culture condition				Solid agar culture condition			
			
		Bruker criteria^a^		Adjusted criteria^b^		Bruker criteria^a^		Adjusted criteria^b^	
					
		Species level	Genus level	Species level	Genus level	Species level	Genus level	Species level	Genus level
Mucorales	23	8 (34.8)	12 (52.2)	20 (87.0)	3 (13.0)	2 (9.0)	11 (47.8)	13 (56.5)	8 (34.8)
*Rhizopus microsporus*	6	3	2	5	1	0	3	3	2
*Rhizopus oryzea*	2	1	1	2	0	0	1	1	1
*Rhizopus azygosporus*	1	0	1	*1*	0	0	0	0	1
*Mucor circinelloides*	3	0	2	2	1	0	1	1	2
*Mucor hiemalis*	1	0	0	0	1	0	0	0	0
*Mucor indicus*	1	0	1	*1*	0	0	0	0	1
*Rhizomucor pusillus*	2	1	1	2	0	1	1	2	0
*Rhizomucor variabilis*	1	0	1	*1*	0	0	1	*1*	0
*Syncephastrum racemosum*	4	3	1	4	0	1	3	4	0
*Lichtheimia ramosa*	2	0	2	2	0	0	1	1	1
*Fusarium*	21	15 (71.4)	5 (23.8)	20 (95.2)	1 (4.8)	5 (23.8)	13 (61.9)	18 (85.7)	3 (14.3)
*Fusarium solani*	9	4	4	8	1	2	5	7	2
*Fusarium moniliforme*	8	8	0	8	0	3	5	7	0
*Fusarium proliferatum*	4	3	1	4	0	0	3	3	1
Others	8	7 (87.5)	1 (12.5)	8 (100)	0	1 (12.5)	6 (75.0)	7 (87.5)	1 (12.5)
*Scedosporium apiospermum*	4	4	0	4	0	1	3	4	0
*Paecilomyces lilacinus*	3	2	1	3	0	0	2	2	1
*Paecilomyces variotii*	1	1	0	1	0	0	1	1	0
Total	52	30 (57.7)	18 (34.6)	48 (92.3)	4 (7.7)	8 (15.4)	30 (57.7)	38 (73.1)	12 (23.1)

**Mucor hiemalis* (*n* = 1), *M. indicus* (*n* = 1), *Rhizomucor variabilis* (*n* = 1), and *Rhizopus azygosporus* (*n* = 1) lack reference spectra in the Bruker library. Numbers in italic and underlined refer to isolates misidentified at the species level. ^*a*^Bruker criteria for the species level is a score ≥2.0; genus-level score is 1.7–1.999. ^*b*^Adjusted criteria for the species level is a score ≥1.7; genus-level score is 1.4–1.699.*

### AFST

The distributions of MICs and MECs of 52 isolates of non-*Aspergillus* molds against six antifungals by the CLSI method are shown in Table 2. For AMB, almost all the tested isolates (51/52, 98.1%) from different species had MICs of ≤ 2 μg/mL except one isolate of *Fusarium solani* that had a higher AMB MIC of 4 μg/mL. In particular, Mucorales had lower MICs of ≤1 μg/mL to AMB in this study. The MICs distribution of triazoles among the tested molds was wide, with range of 0.12 to >16 μg/mL. In Mucorales, 95.7% (22/23) of isolates had MICs of >2 μg/mL to VOR and most of them even were observed with extreme values of >16 μg/mL. As to ITR and POS, 47.8% (11/23) and 34.8% (8/23) of Mucorales isolates had MICs of >2 μg/mL, respectively. In *Fusarium*, 42.9% (9/21) of isolates had MICs of >2 μg/mL to VOR and only 19.0% (4/21) to POS, while there were 42.9% (9/21) of isolates with MICs of >16 μg/mL to ITR. *Scedosporium apiospermum* and *Paecilomyces* isolates hold comparatively diverse susceptibility profiles against triazoles, even with the small number of isolates involved in this study. For echinocandins, as Mucorales and *Fusarium* are widely known to be innately resistant to this class of agents, and *Scedosporium* is somehow less susceptible to echinocandins, high MECs of >8 μg/mL against CAS and MF were observed among these isolates in this study.

**TABLE 2:** Distribution of the median minimum inhibitory concentrations (mMICs)/median minimum effective concentrations (mMECs) and ranges of 6 antifungal agents against 52 isolates of non-*Aspergillus* molds as determined by the CLSI M38-A2 method (unit: μg/mL).

Species	*n*	CLSI M38-A2					
		
		AMB	ITR	VOR	POS	CAS	MF
Mucorales	23	0.5 (0.03–1)	4 (0.25 to >16)	>16 (2 to >16)	0.5 (0.12 to >16)	>8	>8
*Rhizopus microspores*	6	0.5	8	8	0.5	>8	>8
*Rhizopus oryzea*	2	0.5	>16	>16	0.25	>8	>8
*Rhizopus azygosporus*	1	0.5	2	8	0.5	>8	>8
*Mucor circinelloides*	3	0.12	>16	>16	>16	>8	>8
*Mucor hiemalis*	1	0.25	>16	>16	8	>8	>8
*Mucor indicus*	1	0.25	>16	>16	>16	>8	>8
*Rhizomucor pusillus*	2	0.12	0.25	>16	0.25	>8	>8
*Rhizomucor variabilis*	1	0.12	>16	>16	4	>8	>8
*Syncephastrum racemosum*	4	0.25	0.5	8	0.25	>8	>8
*Lichtheimia ramose*	2	1	0.5	>16	0.5	>8	>8
*Fusarium*	21	2 (0.5–4)	4 (0.5 to > 16)	2 (1 to >16)	1 (0.12 to >16)	>8	>8
*Fusarium solani*	9	2	>16	8	1	>8	>8
*Fusarium moniliforme*	8	2	2	2	0.5	>8	>8
*Fusarium proliferatum*	4	1	>16	4	1	>8	>8
Others	8	1 (0.25–1)	1 (0.25 to >16)	1 (0.5 to >16)	0.5 (0.5–1)	4 (≤0.008 to >8)	0.5 (≤0.008 to >8)
*Scedosporium apiospermum*	4	1	1	0.5	0.5	4	1
*Paecilomyces lilacinus*	3	0.25	0.25	1	0.5	≤0.008	≤0.008
*Paecilomyces variotii*	1	0.5	0.06	>8	0.03	4	≤0.008
Total	52	1 (0.03–4)	1 (0.25 to >16)	4 (0.5 to >16)	0.5 (0.12 to >16)	>8 (≤0.008 to >8)	>8 (≤0.008 to >8)

### Agreement Between the CLSI Method and Sensititre YeastOne

The essential agreement (EA) data between the CLSI method and the Sensititre YeastOne on antifungal susceptibility determination of non-*Aspergillus* molds are presented for each genus–drug combination in Table 3. EA values were >90% for the majority of genus–drug combinations; however, the total EA between the CLSI and Sensititre YeastOne to AMB was 84.6%, because the MIC values obtained by the Sensititre YeastOne were generally higher than the CLSI ones especially in Mucorales. For triazoles, acceptable EA values of >90% were reached between those two methods for all the three tested drugs. EA values for echinocandins were excellent in this study, at 100%.

**TABLE 3:** Essential agreement between the CLSI M38-A2 and the Sensititre YeastOne YO10 on antifungal susceptibility testing of non-*Aspergillus* molds (%).

Species	*n*	AMB		ITR		VOR		POS		CAS		MF	
							
		±1 dil.	±2 dil.	±1 dil.	±2 dil.	±1 dil.	±2 dil.	±1 dil.	±2 dil.	±1 dil.	±2 dil.	±1 dil.	±2 dil.
Mucorales	23	10 (43.5)	16 (69.6)	17 (73.9)	22 (95.7)	21 (91.3)	21 (91.3)	15 (65.2)	21 (91.3)	23 (100)	23 (100)	23 (100)	23 (100)
*Fusarium* spp.	21	19 (90.5)	21 (100)	16 (76.2)	19 (90.5)	14 (66.7)	19 (90.5)	20 (95.2)	20 (95.2)	21 (100)	21 (100)	21 (100)	21 (100)
*Paecilomyces* spp.	4	3 (75.0)	4 (100)	4 (100)	4 (100)	2 (50.0)	4 (100)	4 (100)	4 (100)	3 (75.0)	4 (100)	4 (100)	4 (100)
*Scedosporium apiospermum*	4	2 (50.0)	3 (75.0)	2 (50.0)	3 (75.0)	3 (75.0)	3 (75.0)	2 (50.0)	4 (100)	4 (100)	4 (100)	4 (100)	4 (100)
Total	52	34 (65.4)	44 (84.6)	39 (75.0)	48 (92.3)	40 (76.9)	47 (90.4)	41 (78.8)	49 (94.2)	51 (98.1)	52 (100)	52 (100)	52 (100)

*±1 dil. indicates that MIC/MEC values obtained with the two methods fell within 1 dilution. ±2 dil. indicates that MIC/MEC values obtained with the two methods fell within 2 dilutions.*

## Discussion

Identification and antifungal susceptibility testing of non-*Aspergillus* molds are rarely performed as routine tests in most microbiology laboratories. In this study, we identified 52 isolates of non-*Aspergillus* molds from clinical samples through molecular sequencing analysis and MALDI-TOF MS. According to the Bruker MALDI-TOF MS recommended procedure, 57.7% (30/52) of isolates cultured in broth could be identified to the species level with scores of ≥2.0. In contrast, for isolates directly grown on solid agar media (e.g., SDA), a widely used culture method in microbiological laboratories, only 15.4% (8/52) of isolates could achieve species-level identification. This discrepancy of the identification capacities of the Bruker MALDI-TOF MS might be attributed to the different elements grown under those two culture methods, as short-term broth culture mainly yielded mycelia while solid agar culture generated abundant conidia and hyphea within a medium-sized colony. A previous study indicated that young colonies and mature colonies of the identical mold isolate could present some obvious differences in MS spectra (Alanio et al., 2010). It is supposed here that MS spectra of non-*Aspergillus* molds from colonies on solid agar might not be well matched with the reference spectra in the Bruker Filamentous Fungi Library created with the mycelia from liquid broth culture. A short incubation time of about 2 days and sampling of the front hyphea from the young colonies will be favorable for the identification of non-*Aspergillus* molds grown on solid agar media by the Bruker MALDI-TOF MS system.

After lowering the species-level identification COV from ≥2.0 to ≥1.7, the species-level identification rate was increased significantly to 73.1% (38/52) for solid agar–cultured isolates and only one isolate (2.6%, 1/38) was misidentified at the species level, which is comparable with the rate (57.7%) of the Bruker MALDI-TOF MS recommended parameters, namely the broth culture of isolates and the standard COV of ≥2.0. Most of the remaining isolates that failed to be identified at the species level under the adjusted COV, due to the lack of the species-specific reference spectra in the Bruker database, were correctly identified at the genus level. Many studies have established in-house databases expanded with the rare species lacking reference spectra in the Bruker commercial database and improved the identification performance of MALDI-TOF MS remarkably. The mass spectrometry identification (MSI) platform is an outstanding public database featured with the online MALDI-TOF MS identification application and an extensive in-house reference database comprising 938 fungal species (Normand et al., 2017a; Stein et al., 2018; Dupont et al., 2019; Imbert et al., 2019). Notice should be paid that the sample preparation should be in accordance with the recommended method when using these public databases.

Besides the optimization of the comprehensive databases and the interpreting COVs, there were other studies concerning the simplification of the conventional protein extraction method in MALDI-TOF MS identification of filamentous fungi. Most of them have applied the bead grinding procedure plus a short incubation time, with the extraction solution containing formic acid and acetonitrile in one step, which provided labor- and time-saving advantages without increasing the misidentification rates (Luethy and Zelazny, 2018). Attention does need to be paid to biosecurity, especially when performing the bead-based grinding of molds.

In spite of the absence of authorized clinical breakpoints or epidemiological COV for antifungal susceptibility testing of non-*Aspergillus* molds, the antifungal susceptibility results could provide a certain guide for clinical treatment. In this study, the antifungal susceptibilities of 52 isolates of non-*Aspergillus* molds were analyzed by the reference CLSI broth microdilution method, and some underlying profiles were detected even with the limited isolates of each species available for testing. For Mucorales, all isolates presented lower MICs of ≤1 μg/mL to AMB, which suggests good treatment effects according to the suggestion that a cut-off of 0.5 μg/mL for AMB among Mucorales is associated with better outcomes (Lamoth et al., 2016). The tested triazole agents have various susceptibilities, with MICs range from 0.12 to >16 μg/mL for the non-*Aspergillus* molds isolates in this study, and these diverse susceptibility profiles could be observed in isolates from the same species, which supports the importance of testing each individual isolate to guide clinical therapy. Almost all the Mucorales isolates had MICs of >16 μg/mL to VOR and relatively higher MICs of >2 μg/mL to ITR and POS, indicating the difficulty in treatment of mucormycosis. The newly developed extended-spectrum triazole isavuconazole (ISA) is recommended as a salvage therapy of invasive mucormycosis, even the extreme MICs of >16 μg/mL were also detected in some isolates according to the previous research (Miceli and Kauffman, 2015).

A rapid test of the intended antifungal susceptibility of a pathogenic fungus will be very helpful in patient treatment. Some MALDI-TOF MS–based assays offered new perspectives in defining the susceptibility pattern of *Candida* in a fast way, while controversy remains when it comes to molds such as *Aspergillus* (Gitman et al., 2017; Delavy et al., 2019). Since the labor-saving and commercially available Sensititre YeastOne panel holds the same operating principle as the CLSI M38-A2 method in antifungal susceptibility testing except for the endpoint reading patterns, the possibility of determining susceptibility of non-*Aspergillus* molds by Sensititre YeastOne has been discussed. In our study, the essential agreements within 2 dilutions between the CLSI M38-A2 and Sensititre YeastOne results on triazoles and echinocandins against non-*Aspergillus* molds were favorable, with rates of >90%, which met the given criterion. For AMB, the EA rate was lower, 84.6%, between the two methods because Sensititre YeastOne gave higher AMB MICs than the CLSI method, and this phenomenon has been discovered in another study on *Aspergillus* (Wang et al., 2018). In the matter of providing suggestive clues for the clinical treatment of infection with non-*Aspergillus* molds, the Sensititre YeastOne system could be competent, especially when information on the triazole susceptibility is required.

## Conclusion

The Bruker MALDI-TOF MS has favorable performance in identification of non-*Aspergillus* molds directly from young colonies on solid agar media with the adoption of proper adjusted interpretation criteria and the additionally expanded MS database. The Sensititre YeastOne YO10 panel is a simple and reliable alternative to the reference broth microdilution method to determine the antifungal susceptibilities of non-*Aspergillus* molds.

## Data Availability Statement

All datasets generated for this study are included in the article/supplementary material.

## Author Contributions

YL, HW, and Y-CX designed the experiments. YL, XH, and J-JH performed the experiments and analyzed the data. YL, Y-CX, and P-CW participated in the writing of the manuscript. YL, HW, XH, J-JH, P-CW, and Y-CX read and approved the final version of the manuscript.

## Conflict of Interest

The authors declare that the research was conducted in the absence of any commercial or financial relationships that could be construed as a potential conflict of interest.

## Footnotes

1 http://blast.ncbi.nlm.nih.gov/Blast.cgi

2 http://www.mycobank.org/
